# Rab11A-Controlled Assembly of the Inner Membrane Complex Is Required for Completion of Apicomplexan Cytokinesis

**DOI:** 10.1371/journal.ppat.1000270

**Published:** 2009-01-23

**Authors:** Carolina Agop-Nersesian, Bernina Naissant, Fathia Ben Rached, Manuel Rauch, Angelika Kretzschmar, Sabine Thiberge, Robert Menard, David J. P. Ferguson, Markus Meissner, Gordon Langsley

**Affiliations:** 1 Hygieneinstitut, Department of Parasitology, University Hospital Heidelberg, Heidelberg, Germany; 2 Laboratoire de Biologie Cellulaire Comparative des Apicomplexes, Department of Infectious Diseases, Institut Cochin, Inserm U567, CNRS UMR 8104, Faculté de Médecine Paris V – Hôpital Cochin, Paris, France; 3 Unité de Biologie et Génétique du Paludisme, Institut Pasteur, Paris, France; 4 Nuffield Department of Pathology, University of Oxford, John Radcliffe Hospital, Oxford, United Kingdom; Washington University School of Medicine, United States of America

## Abstract

The final step during cell division is the separation of daughter cells, a process that requires the coordinated delivery and assembly of new membrane to the cleavage furrow. While most eukaryotic cells replicate by binary fission, replication of apicomplexan parasites involves the assembly of daughters (merozoites/tachyzoites) within the mother cell, using the so-called Inner Membrane Complex (IMC) as a scaffold. After *de novo* synthesis of the IMC and biogenesis or segregation of new organelles, daughters bud out of the mother cell to invade new host cells. Here, we demonstrate that the final step in parasite cell division involves delivery of new plasma membrane to the daughter cells, in a process requiring functional Rab11A. Importantly, Rab11A can be found in association with Myosin-Tail-Interacting-Protein (MTIP), also known as Myosin Light Chain 1 (MLC1), a member of a 4-protein motor complex called the glideosome that is known to be crucial for parasite invasion of host cells. Ablation of Rab11A function results in daughter parasites having an incompletely formed IMC that leads to a block at a late stage of cell division. A similar defect is observed upon inducible expression of a myosin A tail-only mutant. We propose a model where Rab11A-mediated vesicular traffic driven by an MTIP-Myosin motor is necessary for IMC maturation and to deliver new plasma membrane to daughter cells in order to complete cell division.

## Author Summary

Apicomplexan parasites are unusual in that they replicate by assembling daughter parasites within the mother cell. This involves the ordered assembly of an Inner Membrane Complex (IMC), a scaffold consisting of flattened membrane cisternae and a subpellicular network made up of microtubules and scaffold proteins. The IMC begins to form at the onset of replication, but its maturation occurs at the final stage of cytokinesis (the last step during cell division) upon the addition of motor (glideosome) components such as GAP45 (Glideosome Associated Protein), Myosin A (MyoA), and Myosin-Tail-Interacting-Protein (MTIP, also known as Myosin Light Chain 1) that are necessary to drive the gliding motility required for parasite invasion. We demonstrate that Rab11A regulates not only delivery of new plasmamembrane to daughter cells, but, importantly, also correct IMC formation. We show that Rab11A physically interacts with MTIP/MLC1, implicating unconventional myosin(s) in both cytokinesis and IMC maturation, and, consistently, overexpression of a MyoA tail-only mutant generates a default similar to that which we observe upon Rab11A ablation. We propose a model where Rab11A-mediated vesicular traffic is required for the delivery of new plasma membrane to daughter cells and for the maturation of the IMC in order to complete cell division.

## Introduction

Cytokinesis, the final step during cell division, has been extensively studied in eukaryotes. Whereas in animal cells cytokinesis is dependent on the formation of an actin/myosin-based contractile ring that forms in the middle of the anaphase spindle [1],[2], in plants the phragmoplast (a specialised cytoskeleton scaffold) of microtubules and microtubule-associated proteins delivers vesicles to the equatorial plate. Upon fusion, these vesicles form the new plasma membrane (for a review see [3]). In contrast, replication of apicomplexan parasites involves the internal budding of multiple daughter parasites inside a single mother (2 in case of *T. gondii*, circa 32 in case of blood stage *Plasmodium spp.*).

Although the temporal organization of morphological changes of organelles during replication of apicomplexan parasites has been well documented by several groups [4],[5] to date almost nothing is known about the molecular mechanisms involved.

During replication a scaffold for the assembly of daughter parasites is built that is known as the Inner Membrane Complex (IMC). As daughter parasites grow they acquire a complete set of organelles, via *de novo* synthesis (i.e. micronemes and rhoptries), or replication/segregation (i.e. ER, Golgi, Mitochondria and Apicoplast) [6],[7]. At the final stage of cell division parasites are believed to simply bud from the mother cell, picking up plasma membrane and leaving unwanted material behind in a residual body [7].

The IMC and the underlying subpellicular microtubules appear to have a central role during these processes, since microtubule-modifying drugs, such as Oryzalin, effectively block organelle segregation and daughter cell budding [7],[8]. In contrast, treatment of parasites with cytochalasin D (CD) results in the formation of large residual bodies, whereas segregation of organelles is not affected. This might indicate a role for actin and myosin during the late stages of replication [8]. Interestingly, enlarged residual bodies have also been identified in parasites over-expressing an unconventional myosin called MyoB [9]. Furthermore, the actin-related protein ARP1 has been recently implicated in IMC formation in *T. gondii* [10].

Several components of the IMC have been identified in apicomplexan parasites such as putative scaffolding proteins [11],[12]. It is noteworthy that ablation of IMC1a, an IMC1 isoform that is expressed during sporogony in *P. berghei* indicates that a structurally intact IMC is required for mechanical stability of sporozoites and gliding motility. However, replication and organelle segregation do not appear to be affected [13]. Therefore, it is possible that the IMC is necessary to provide stability to the parasite to withstand mechanical stress, for example during gliding motility. Importantly, the glideosome (a motor complex required for gliding motility) is anchored to the IMC. Assembly of the glideosome occurs in two steps. The Gliding Associated Protein 50 (GAP50) directly inserts within the IMC, as an integral membrane protein and is believed to act as an anchor for the remaining components (MyoA, MTIP/MLC and GAP45), which only associate with the IMC of mature daughter parasites, at the final stage during the replication of the parasite [14].

The small G-protein Rab11A is conserved in eukaryotes and it has been shown to play a key role in regulating trafficking of certain plasma membrane receptors through recycling endosomes [15]. In addition, Rab11A has been demonstrated to be required for delivering plasma membrane to the cleavage furrow in animal cells [16],[17] and to be localised at the division plane of plant cells [18], indicating a conserved function during cell division. Apicomplexan Rab11A was first described in *P. falciparum* [19] and subsequently shown to be expressed in asexual blood stage parasites [20]. Rab11A was also found to be associated with the rhoptries of *Toxoplasma* [21] and we recently demonstrated that expression of a dominant negative version of Rab11A in *T. gondii* is deleterious for the parasite resulting in reduced growth [22].

In this paper, we analyse in detail the function of Rab11A and provide a mechanistic insight into the late steps of cell division and assembly of the glideosome in apicomplexan parasites. Inducible ablation of Rab11A function generates a block in late stages of cell division and leads to incomplete maturation of the IMC, consistent with a role for the IMC in cell division. Genetic experiments performed in *P. berghei* argue that the Rab11A protein performs an essential function for parasite development in red blood cells. We propose a model, where Rab11A-mediated vesicular traffic driven by an MTIP/Myosin motor is required for correct assembly of the IMC and generation of daughter parasites at the final stage of daughter cell assembly that corresponds to cytokinesis.

## Results

### Rab11A is highly conserved in apicomplexan parasites

The first parasite Rab11A sequence to be described was that of *P. falciparum* in 1996 [19] and like other eukaryotes, *P. falciparum* parasites also have a second *rab11* gene (for all accession numbers, see Table S1) coding for Rab11B [20],[23]. Subsequently, it was shown that Rab11A was expressed in *P. falciparum*-infected red blood cells [20]. Rab11 sequences from two other apicomplexan parasites have since been described with one from *Babesia gibsoni* [24] and the other from *T. gondii* [21]. Similar to *Plasmodium spp.*, both *Babesia* and *Toxoplasma* encode both Rab11A and Rab11B [25]. Only Rab11A has been described as being rhoptry-associated (rhoptries are invasion associated organelles of the secretory system) in *T. gondii* [21]. As the genome sequences of both *Theileria* and *Cryptosporidia* are available we compared the different parasite Rab11A sequences with those of Man and yeast to demonstrate the high degree of conservation of this GTPase amongst apicomplexan parasites (Figure S1). Given this level of conservation (e.g. circa 76% identity and 87% similarity) amongst parasites it seems reasonable to suppose that Rab11A performs similar functions in both *Plasmodium spp.* and *Toxoplasma* and that by dissecting and comparing its function in the two *Apicomplexa* one ought to gain insights into Rab11A-mediated processes in this group of medically important parasites.

### Rab11A has a dynamic distribution throughout parasite development in red blood cells

**Figure 1:**
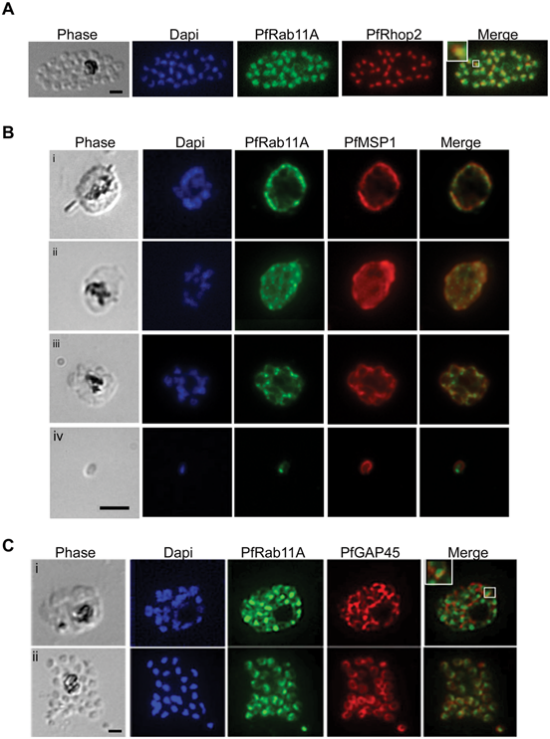
PfRab11A has a dynamic localisation. (A–C) Throughout the erythrocytic stage of parasite development the localisation of PfRab11A changes. (A) Rhoptry localisation was confirmed in mature schizonts by immuno-staining infected blood smears with anti-PfRab11A and co-localisation (enlargement shown boxed in merge) demonstrated with anti-PfRhopH2 (rhoptry specific marker) antibodies. The bright-field image shows a mature schizont with the nuclei stained with Dapi. (B) PfRab11A co-localises partially with PfMSP1 (plasma membrane specific marker) in trophozoites (i, ii) and schizonts (iii). The merged images show co-localisation of the two proteins, giving single dots. In merozoites (iv), the localisation of PfMSP1 and PfRab11A is now distinct with PfRab11A having an apical localisation slightly under the plasma membrane decorated by PfMSP1. (C) The first two panels show the bright-field (phase) and Dapi, respectively. The last panel in all images shows an overlay. In young (i) and segmented (ii) schizonts, PfRab11A partially co-localises with PfGAP45 (Glidosome Associated Protein 45 used as an Inner Membrane Complex specific marker). In all cases, the black scale bar in the first panel of each set of images represents 5 µm.

Rab11A has been shown to be rhoptry-associated in *T. gondii* [21], which might indicate a role for this GTPase in regulating vesicular traffic to the rhoptries, and/or in release of rhoptry contents in the *Apicomplexa*. However, in *P. falciparum*-infected red blood cells Rab11A expression could also be detected before rhoptries are formed [20], suggesting that its sub-cellular localisation could be dynamic during intra-erythrocyte development. To analyse in more detail the sub-cellular distribution of Rab11A we performed confocal microscopy using anti-PfRab11A antibodies and *P. falciparum*-infected erythrocytes harbouring parasites at different stages of development (Figure 1). First, to confirm that in *P. falciparum*, as in *T. gondii*, Rab11A is rhoptry-associated we co-stained with an antibody to the rhoptry specific protein Rhop2 [26] and found significant co-localisation (see enlargement shown boxed in merge) between Rab11A in late stage schizonts, as rhoptries are being formed (Figure 1A). Next, we examined Rab11A distribution now comparing it to that of the merozoite surface protein 1 (MSP1) from trophozoites through to schizonts and merozoites and noticed that the two proteins show a dynamic pattern of distribution with spots of clear co-localisation in young schizonts that becomes quite distinct in merozoites (Figure 1B). In merozoites as expected MSP1 decorates the surface [27],[28], whereas Rab11A now appears to lie just under the plasma membrane with an apical concentration typical of rhoptries. We also compared the sub-cellular localisation of Rab11A and the Glidosome Associated Protein 45 (GAP45) and observed double-positive vesicles (boxed area) consistent with the notion that their association is dynamic and that GAP45 might be delivered to the IMC via Rab11A-mediated pathway (Figure 1C). Once merozoites are formed, Rab11A is localised at the rhoptries and GAP45 at the IMC just under the plasma membrane (Figure 1A, B_iv_, and C_ii_).

**Figure 2:**
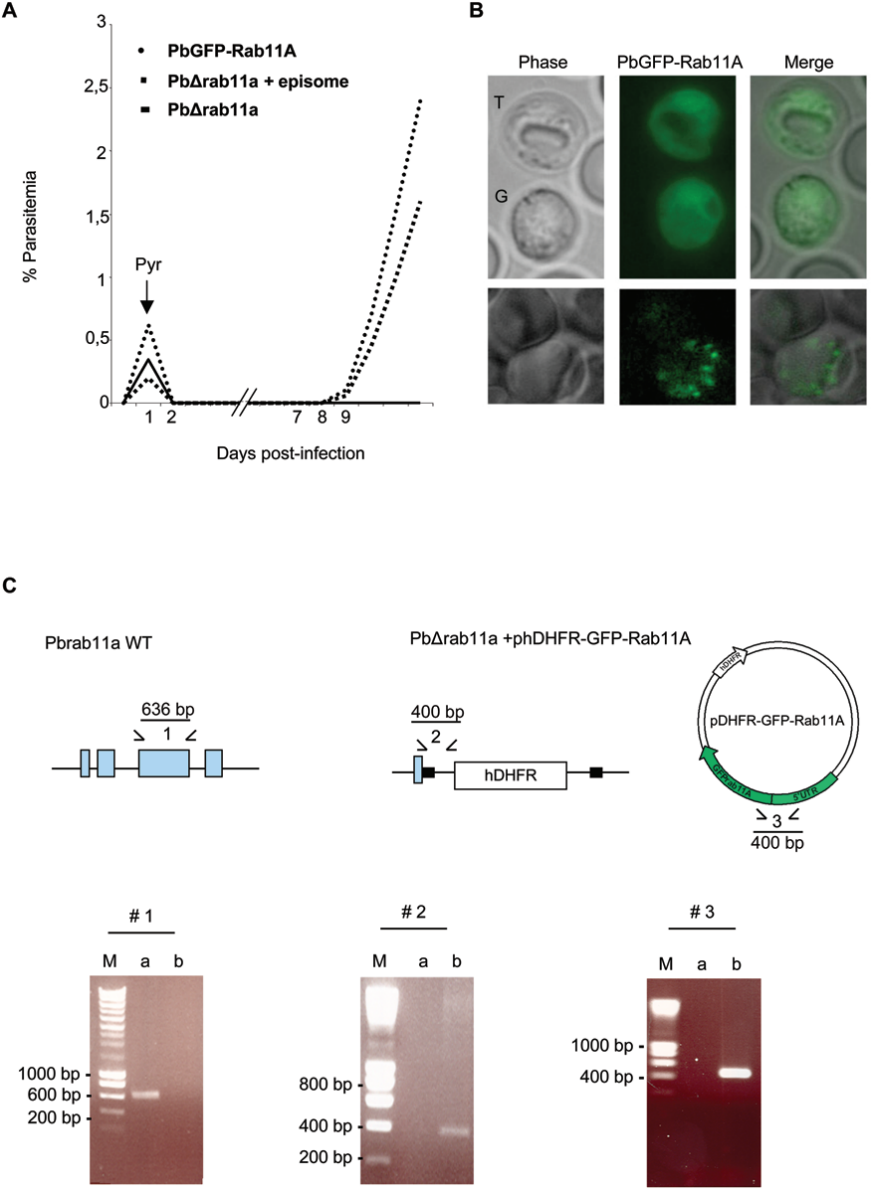
The *P. berghei rab11a* gene is essential for growth in red blood cells. (A) Growth comparison of transgenic parasites. To disrupt the *rab11a* locus, *P. berghei* merozoites were electroporated with the HindIII/EcoRI-linearized replacement plasmid containing 5′- and 3′-untranslated regions (black boxes in C) of *rab11a* and the human *dhfr* selectable marker (hDHRF); electroporated merozoites were subsequently injected into young mice. Drug selection of transfectants was by pyrimethamine (Pyr) addition to the drinking water 24 h post-transfection. No PbDHΔ11a disrupted parasites were recovered, only transfection of the linearized plasmid together with phDHFR-GFPRab11A vector (episome) generated transgenic parasites. The growth of rescued parasites harbouring the PbΔrab11a+episome is slower compared to parasites harbouring just episomal PbGFP-Rab11A. (B) PbRab11A localisation in trophozoites (T) and gametocytes (G) appears cytoplasmic, whereas in schizonts (lower panel) it appears more vesicular rhoptry-like. (C). Replacement-specific PCR analysis: A wild type specific PCR using primer combinations indicated by (1) was performed to confirm the disruption of the endogenous gene (exons shown in blue) in the clonal rescued parasite population. Confirmation of integration into the *Pbrab11a* locus and the presence of the episome were achieved by specific primer combinations (2 and 3), which detect the recombinant locus and the episome in transgenic parasites. The products run on 1% (wt/vol) agarose gel were stained with ethidium bromide. The molecular sizes are indicated in base pair (bp). The expected size of the respective PCR product is indicated in the diagram. M: marker; a and b: DNA isolated from wt and PbΔrab11a (+episome) parasites, respectively.

To further analyse Rab11A sub-cellular distribution during development within red blood cells, we generated by single crossover recombination at the 5′-UTR of the *rab11a* gene *P. berghei* transgenic parasites expressing GFP-Rab11A (Figure S2). We choose to insert the tagged copy upstream of the endogenous *rab11a* gene, so as to leave it intact, as we suspected that deletion of Rab11A function would be lethal and indeed, this turned out to be the case (see below). We also took the precaution of using *rab11a* 5′- and 3′-UTRs to drive expression so as to increase the probability that transgene expression is close to endogenous levels. Rab11A was also N-terminally GFP-tagged, so as not to interfere with correct geranylageranylation of its C-terminus [29],[30]. Proof that GFP-Rab11A is functionally active came from its ability to “rescue” parasites lacking the endogenous *rab11a* gene, when the tagged version was expressed off an episome (Figure 2A).

We obtained parasites expressing GFP-Rab11A throughout the asexual life cycle (Figure 2B), confirming both the IFA studies above, our RT-PCR results (not shown) and microarray data (PF13_0119) available at PlasmoDB. Interestingly, we found a rather diffuse, vesicular localisation of GFP-Rab11A in trophozoites and gametocytes, whereas in schizonts, a clear apical-like localisation is obvious (Figure 2B) that closely resembles the pattern observed in fixed *P. falciparum* schizonts (Figure 1). Therefore, we conclude that Rab11A has a dynamic localisation during intra-erythrocytic development of *Plasmodium* parasites associating with rhoptries only after their biogenesis. Thus, the changing distribution of Rab11A could indicate that the GTPase could be performing non-rhoptry associated functions during development of the parasite within red blood cells.

### Rab11A is essential for parasite development within host cells

Our ability to generate GFP-Rab11A transgenic parasites by single crossover recombination indicated that the *rab11a* locus was susceptible to genetic manipulation. We turned therefore, to reverse genetics to determine if Rab11A plays an essential function and interrupted the *rab11a* locus in *P. berghei* parasites via double crossover homologous recombination (Figure S2). Since loss of the single *rab11a* allele in haploid *P. berghei* blood stage parasites appeared lethal (no KO parasites obtained), we performed an “episome rescue” [31], complementing loss of the nuclear encoded Rab11A by providing the essential function with GFP-Rab11A encoded on an episome. PCR was used to demonstrate loss of the nuclear copy (Figure 2C) and the KO parasites grew due to functionally active GFP-Rab11A being provided *in trans* off the circular episome (Figure 2A).

### Rab11A physically interacts with MTIP, a crucial component of the glideosome

**Figure 3:**
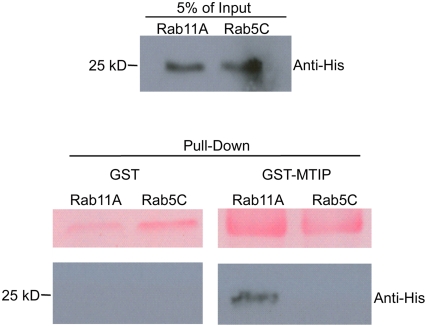
PfRab11A associates with PfMTIP *in vitro*. The purified recombinant proteins PfRab11A-His and PfRab5C-His (top panel) were mixed with GST or GST–MTIP (bottom panel). Anti-His antibodies (Santa Cruz) were used to detect pulled-down His-tagged Rab protein and only PfRab11A associates with PfMTIP. The upper panel of the pull down correspond to Ponceau S stain of the membrane to demonstrate loadings of GST and GST-MTIP, respectively.

Rab11A-mediated transport in Man and yeast can be provided by interaction with unconventional myosin V [32], reviewed in [33]. However, apicomplexan parasites lack a recognisable orthologue of myosin V, but nonetheless, have several unconventional myosins (10 in *T. gondii* and 6 in *Plasmodium*) [34],[35]. However, only a few myosin light chains can be identified in the respective genomes (only one in *Plasmodium* termed MTIP). Although MLC1/MTIP has been initially described as the light chain for MyoA [36],[37], it is plausible that several other myosins require MLC1/MTIP in order to function as a motor proteins. Since we found partial co-localisation of Rab11A with components of the glideosome (GAP45), we asked, whether Rab11A associates with MTIP and via this association connects to an unconventional myosin to derive motif force for vesicle transport. To this end, we performed a series of pull-down experiments using GST-tagged MTIP and his-tagged Rab11A. Recombinant *P. falciparum* Rab5C, GST-only (Figure 3) and Rab7 (not shown) were used as (negative) controls. Only Rab11A was found to specifically interact with MTIP. We found that approximately 5% of the original input was detected in the pull down, indicating a transient interaction between Rab11A and MTIP (Figure 3). This supports the notion that an unconventional Myosin/MTIP motor drives a Rab11A-mediated transport.

### Expression of Rab11A_(N126I)_ results in a defect in late step during cell division

**Figure 4:**
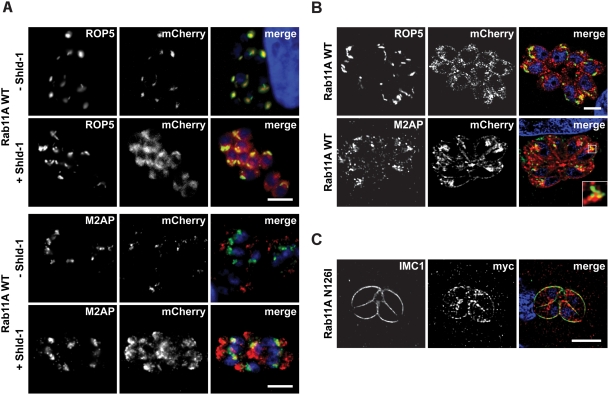
Overexpression of the wild type and dominant-negative Rab11A in *T. gondii*. (A) Regulation of ddFKBP-mCherry-Rab11A_wt_ expression in stable transfected parasites. Parasites were inoculated on HFF cells in presence and absence of 1 µM Shld-1 for 16 hours and probed with the indicated antibodies. In the absence of Shld-1, weak background fluorescence can be detected that co-localises with the rhoptry marker Rop5. Overexpression (+Shld-1) of Rab11A_wt_ results in partial accumulation in an endosome-associated compartment as indicated by partial co-localisation with proM2AP [39]. Scale bar: 5 µm. (B) Confocal microscopy of the same parasite strain as in (A). High-resolution microscopy reveals that overexpression of Rab11A results in association with a “vesicular network” to which the rhoptries appear to align. Again a partial co-localisation with proM2AP is evident. Scale bar: 5 µm. (C) Overexpression of dominant negative Rab11A_(N126I)_ N-terminally tagged with ddFKBPmyc results in a different, vesicular localisation that accumulates at the IMC as indicated by partial co-localisation with antibody against IMC1 [57]. Note that no accumulation is evident at the rhoptries (see also Figure S3). Scale bar is 5 µm. The green and red colours in the merged images correspond to the left and middle panels, respectively.

Having established that *rab11a* is an essential gene in *P. berghei*, to gain further insights into potential Rab11A functions we decided to characterise loss of function phenotypes and turned to the ddFKBP-system to induce expression of different versions of Rab11A in *T. gondii* [22]. During the delivery of vesicular material from a donor- to an acceptor-membrane Rabs switch from a GTP-bound to a GDP-bound form via GTP-hydrolysis that is activated by a rabGAP [38]. To analyse Rab11A function we therefore, generated different expression vectors and a dominant-negative (GDP-locked) version harbouring a point mutation in the GTPase domain (N126I). Since expression of N126I has been demonstrated to be deleterious for the parasite, we placed both Rab11A_wt_ and Rab11A_(N126I)_ under control of an N-terminal ddFKBP-myc-tag (in the following only mentioned as ddFKBP), which allows regulation of recombinant protein levels by the inducer Shield-1 (Shld-1) [22]. In addition, we generated parasites expressing mCherry-tagged versions of Rab11A combined with ddFKBP. We confirmed that neither the addition of N-terminal ddFKBP, nor that of mCherry had an influence on the location of Rab11A (data not shown). In absence of the inducer Shld-1 ddFKBP-mCherry tagged wild type Rab11A is rapidly degraded and only a weak background fluorescence can be detected that co-localises with the rhoptry protein 5 (Figure 4A), confirming the established rhoptry location of Rab11A within *Toxoplasma* [21]. Addition of Shld-1 results in stabilisation of the respective ddFKBP-tagged construct and under these conditions we found that Rab11A levels accumulate and can now be readily observed at other compartments distinct from the rhoptries (Figure 4A and 4B) that showed partial co-localisation with the propeptide of the MIC2 associated protein (M2AP), a marker for endosome-associated compartments [39]. We confirmed that over expression of Rab11A_wt_ did not result in a detectable phenotype (data not shown).

As we had previously observed that parasites expressing Rab11A_(N126I)_ show a severe growth defect [22], we now examined in detail parasites, where we ablate Rab11A function by controlled accumulation of trans-dominant negative Rab11A_(N126I)_ and compared the induced phenotype with similarly treated parasites expressing wild type Rab11A. As expected for an inactive (GDP-bound) form [38], we observed a rather diffuse cytosolic location of dominant-negative Rab11A_(N126I)_, with no obvious association with the rhoptries (Figure S3). Interestingly, we found a significant amount associated with a structure similar to the IMC between the forming daughter cells (Figure 4C and Figure S3). We employed different organelle and cytoskeleton markers and although the rhoptry location of Rab11A might suggest a function in rhoptry biogenesis or trafficking of rhoptry proteins, we failed to detect any defects in parasites expressing Rab11A_(N126I)_ (Figure S3). Similarly, no effect on other secretory organelles (micronemes and dense granules) was obvious in this mutant (Figure S3). We also analysed the fate of other organelles during the replication of the parasite (Golgi, apicoplast, mitochondria and nucleus), but failed to detect any defect in segregation/biogenesis (data not shown), indicating that the block in daughter cell division occurs at a late stage [7]. In support of this hypothesis, the formation and elongation of the IMC appeared to be normal during replication, since neither the formation, nor localisation of subpellicular microtubules (Figure S3), nor the scaffolding protein IMC1 appeared to be affected (Figure 4C).

**Figure 5:**
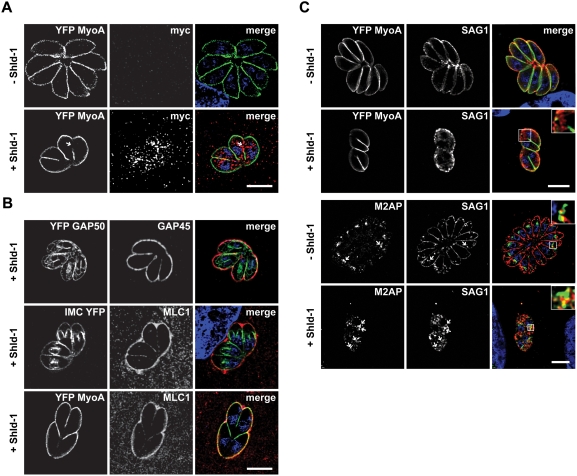
Expression of Rab11A_(N126I)_ results in a specific effect on the formation of the glideosome. Parasites expressing ddFKBPRab11A_(N126I)_ were inoculated on host cells (HFF). After invasion, intracellular parasites were treated with or without 1 µM Shld-1 for 16 hours, before samples were fixed and stained with the indicated antibodies. (A) Parasites co-expressing YFP-MyoA and ddFKBPRab11A_(N126I)_ were analysed. Note that tagged Rab11A_(N126I)_ (as detected with myc-antibodies) specifically accumulates at regions where less YFP-MyoA is detectable within the IMC (arrow). (B). Analysis of further components of the glideosome/IMC in parasites expressing ddFKBPRab11A_(N126I)_. Top: Parasites co-expressing YFP-GAP50 [14] and ddFKBPRab11_(N126I)_. Note that GAP50 can be identified at the IMC of first- and premature second-generation daughter parasites. In contrast, GAP45 (as detected with GAP45 antibodies) can mainly be identified at the IMC of the original mother parasite. Only a faint staining of the first-generation daughter cells is detectable. Middle: Same experiment with parasites co-expressing IMC1-YFP [53] and ddFKBPRab11A_(N126I)_. The localisation of MLC1 is restricted to the first-generation mother cell, whereas IMC1 can be detected at the IMC of first- and second-generation daughter cells. Bottom: Same parasites as in A). Both, MyoA and MLC1 show mainly staining of the mother parasite. (C) Expression of Rab11A_(N126I)_ results in intracellular accumulation of SAG1. Upper panels: Parasites co-expressing YFP-MyoA and ddFKBPRab11A_(N126I)_ were stained using monoclonal SAG1 antibodies. Whereas in the absence of Shld-1 parasite replication was evident with SAG1 exclusively detected at the surface, stabilisation of Rab11A_(N126I)_ resulted in intracellular localisation of SAG1. Note that even plasmamembrane-associated SAG1 shows a rather patchy pattern. Lower panels: Parasites expressing ddFKBPRab11A_(N126I)_ have been grown under the same conditions and a co-localisation with antibodies against proM2AP and SAG1 has been performed. Internalised SAG1 partially co-localises with proM2AP positive endosomes (arrows). In the upper corners, an enlarged area of the boxed area is shown. Scale bars: 5 µm. The green and red colours in the merged images correspond to the left and middle panels, respectively.

One of the final steps during parasite replication is the assembly of the glideosome at the IMC of the daughter cells and the motor complex is assembled in two temporally separated steps. Whereas GAP50 is immediately integrated into the IMC, the remaining components (GAP45, MLC/MTIP and MyoA) are believed to associate in the cytosol to form a proto-glideosome and associate with GAP50 only after the final assembly of the daughter cells [14]. When we analysed replicating parasites for association of MyoA with the IMC of daughter parasites, we found that this motor protein is less efficiently associated with the IMC when ddFKBPRab11A_(N126I)_ was stabilised by addition of Shld-1 (Figure 5A). In fact it appeared that MyoA is mainly associated with the IMC of the first generation mother cell, but not with the IMC of subsequent generations (Figure 5A). Interestingly it appeared that Rab11A_(N126I)_ accumulates around areas where less or no association of MyoA with the IMC is evident (see arrow in Figure 5A). We next analysed if a similar phenotype is evident with other components of the glideosome. We inoculated ddFKBPRab11A_(N126I)_ parasites in presence of Shld-1 and analysed maturation of the IMC (Figure 5B). We did not observe any effect on the integration of the early components GAP50 and IMC1 into the IMC of daughter parasites. In contrast both, GAP45 and MLC-1 showed an identical staining pattern as MyoA (Figure 5B). Again Rab11A_(N126I)_ appeared to be concentrated around areas where less GAP45, or MLC1 is associated with the IMC (Figure S3, and data not shown). Together, these results demonstrate that Rab11A regulates an essential step during cell division, after biogenesis of the secretory organelles (micronemes and rhoptries), but before assembly of the motor complex at the IMC.

In animal and plant cells the final stage during cell division is the deposition of new plasma membrane between the daughter cells and Rab11A has been demonstrated to play an important role in this process by directing recycled membrane material to the division plane/furrow [40]. To analyse, if biogenesis of new plasma membrane is required during cytokinesis of *T. gondii*, we followed the location of the major surface antigen SAG1 during replication. We found that parasites expressing Rab11_(N126I)_ show an abnormal location of SAG1. The typical smooth staining pattern of SAG1 at the surface of the parasite appeared to be lost and a rather patchy location at the plasma membrane of the mother was evident (Figure 5C). Importantly, we detected a vesicular signal for SAG1 within the parasite, indicating that SAG1 is not delivered to the surface in absence of functional Rab11A. In fact it appeared that SAG1 partially accumulates close to the endosomal compartments, as evidenced by partial co-localisation with proM2AP (Figure 5C).

Together these data suggest that Rab11A is required for the delivery of vesicles, containing SAG1 and probably other surface proteins, from the endosomal network to the plasmalemma of daughter cells, where new plasma membrane is synthesized, similar to the function described in other eukaryotes [15].

### Over-expression of MyoA-tail results in an analogous phenotype to loss of Rab11A function

**Figure 6:**
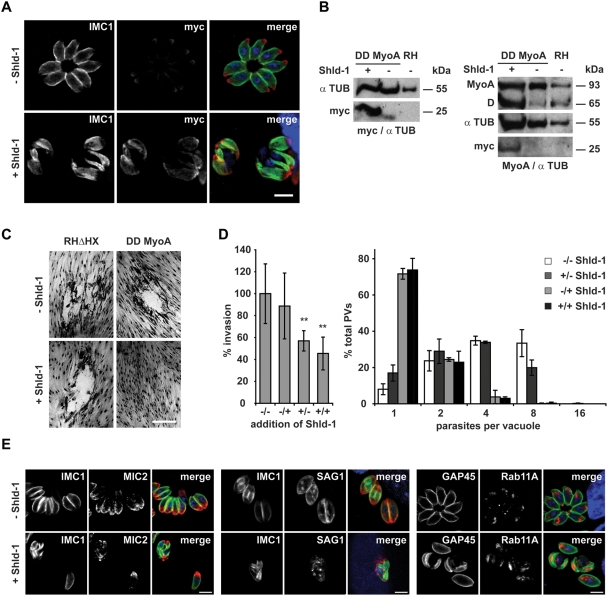
Inducible expression of a dominant negative MyoA results in a similar phenotype as observed for Rab11A_(N126I)_. (A). Stable transfection of wild type parasites with the construct p5RT70ddFKBPMyoA_tail_ allows specific and inducible regulation of ddFKBPMyoA_tail_ in dependence of Shld-1. Parasites were inoculated on HFF cells in the presence and absence of 1 µM Shld-1 and stained with the indicated antibodies. In the absence of Shld-1, weak background levels of ddFKBPMyoA_tail_ can be detected close to the apical pole of the parasite. In the presence of Shld-1, high protein levels can be detected that co-localise with the IMC. Not that these parasites show a very abnormal IMC when compared to non-treated parasites. Scale bar: 5 µm. The green and red colours in the merged images correspond to the left and middle panels, respectively. (B) Immunoblot analysis of parasites stably transfected with p5RT70ddFKBPMyoA_tail_. Parasites were incubated for 5 hours in the presence or absence of Shld-1 before lysates were prepared. Left: Blot was simultaneously probed with myc- and alpha-Tubulin (as loading control) antibodies to analyze Shld-1 dependent regulation of ddFKBPMyoA_tail_. Right: Blot was simultaneously probed with antibodies against MyoA and alpha-Tubulin. Occasionally we detected a degradation product (D) of endogenous MyoA at ∼65 kDa that appears to be more prominent when ddFKBPMyoA_tail_ is stabilized. (C) Stabilization of ddFKBPMyoA_tail_ is deleterious for the parasite. Stable transfected parasites were inoculated on HFF cells in the presence and absence of 1 µM Shld-1 for 6 days before formation of plaques was compared. In the presence of Shld-1, no growth of parasites was detected. Scale bar 20 µm. (D). Invasion and replication analysis of parasites expressing ddFKBPMyoA_tail_. (−/−) parasites without Shld-1 treatment; (−/+) parasites treated with Shld-1 after invasion; (+/−) parasites treated with Shld-1 before invasion and without Shld-1 after invasion; (+/+) parasites constantly kept under Shld-1 treatment. Left: For the invasion assay, the total number of parasitophorous vacuoles was determined. Mean values of 5 (+/− and −/+) and 6 (−/− and +/−) independent experiments ±s.d. are shown. Asterisks indicate significant difference in total invasion compared to parasite strain ddFKBPMyoA_tail_ not treated with Shld-1 before and after invasion (P<0.01, two tailed Student's t-test). Right: For the replication assay the number of parasites per parasitophorous vacuole was determined. Mean values of three independent experiments are shown +/−s.d. (E) Immunofluorescence analysis of parasites stably transfected with p5RT70ddFKBPMyoA_tail_. Parasites were treated with Shld-1 after invasion of the host cell (+) or left untreated (−). 16 hours post invasion, parasites were fixed and analysed with the indicated antibodies. Scale bars: 5 µm. The green and red colours in the merged images correspond to the left and middle panels, respectively.

Given the above demonstrated association between *P. falciparum* Rab11A and MTIP (Figure 3), we surmised that MLC1/MTIP associated with an unconventional myosin might provide motile force for Rab11A-mediated vesicular traffic during cytokinesis. We choose to over-express only the tail of *Toxoplasma* MyoA in an attempt to compete with endogenous myosins (MyoA and possibly other myosins) for formation of functional motor complexes, that require MLC1/MTIP, reasoning that it might result in deregulated myosin function, similar to reports for yeast myosin V [41]. To this end, we generated stable *T. gondii* transfectants expressing just the MyoA-tail fused to ddFKBP and as expected, addition of Shld-1 resulted in its strong accumulation (Figure 6A and 6B). We confirmed in growth assays that over-expression of just the MyoA-tail is deleterious (Figure 6C). Interestingly, as is the case for expression of mutant Rab11A [22], we found a dual phenotype due to expression of MyoA-tail. While expression in extra-cellular parasites resulted in a significant block in invasion (Figure 6D), expression in intracellular parasites caused a complete block of replication with parasites being arrested at the 1–4 cell stage (Figure 6D). We examined next if parasites blocked in replication show a similar phenotype to those ablated for Rab11A function. Indeed, the MyoA-tail-induced block also generates a defect in the correct assembly of the IMC. However, we noticed that IMCs of daughter cells are almost completely collapsed within the mother cell (Figure 6E).

This might indicate that additional myosins that require MTIP/MLC1 for their function is affected. Nonetheless, as in case with Rab11A_(N126I)_ expression, organelle segregation and biogenesis appears to be not affected during replication (Figure 6E, and data not shown), Together, these results suggest that Rab11A and unconventional myosins are functionally linked via their mutual association with MTIP/MLC1 and together they regulate IMC assembly and daughter cell budding.

### Ultrastructural analysis of DN-mutants

**Figure 7:**
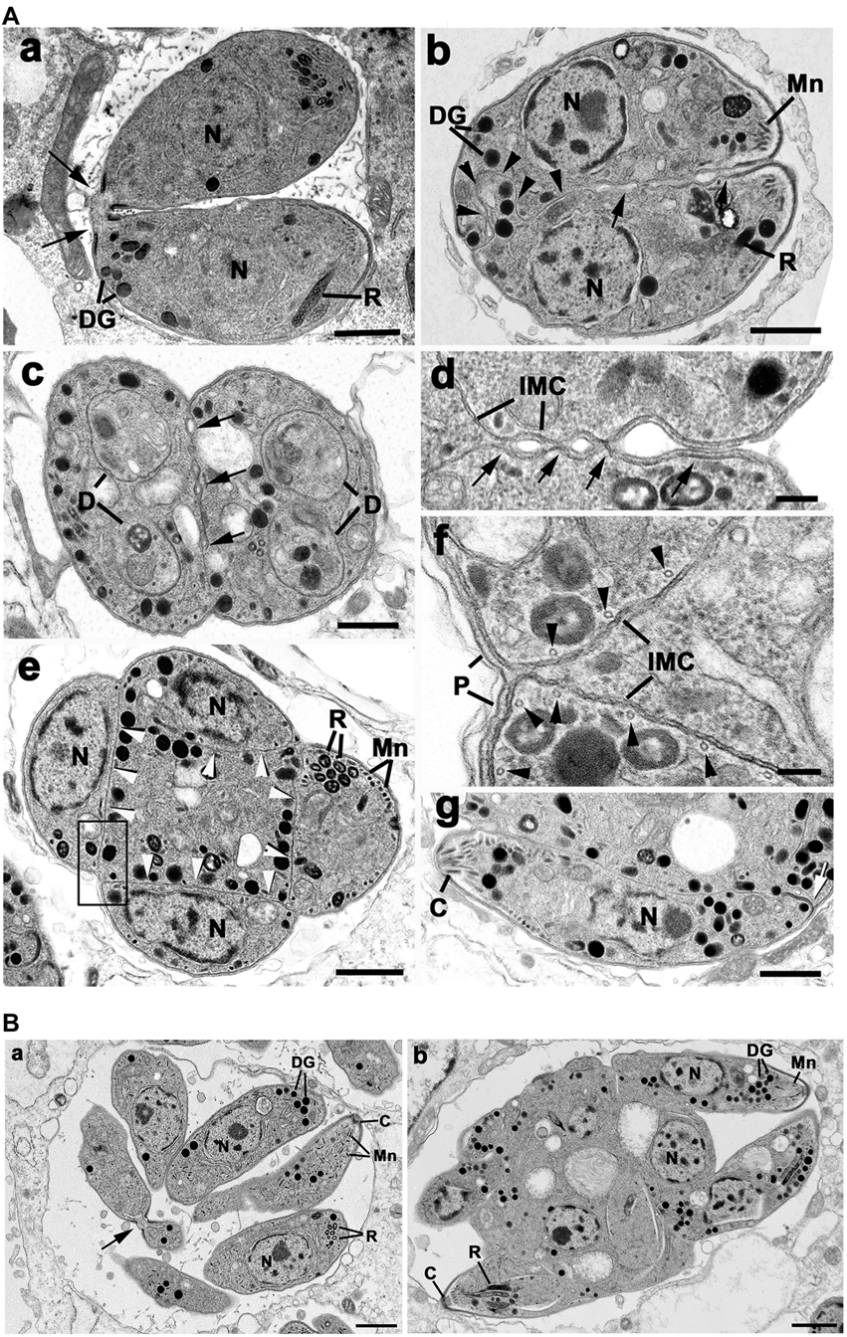
Transmission electron micrographs of parasites expressing ddFKBPRab11A_(N126I)_ treated with or without Shld-1. (A) Transmission electron micrographs of samples not treated with Shld-1 (a) and treated with Shld-1 for 24 hours (b–g). (a) Longitudinal section through a control sample showing two daughters formed by endodyogeny still connected by their posterior end (arrows). Bar is 1 µm. (b) Longitudinal section through a sample treated with Shld-1 illustrates two fully formed daughters incompletely separated along their lateral surface (arrows) and with an irregular IMC towards the posterior (arrowheads). Bar is 1 µm. (c) Cross section through two incompletely separate daughters (arrows) showing the initiation of a second round of endodyogeny with the formation of the two IMC of the second generation of daughters (D) within each first-generation daughter. Bar in 1 µm. (d) Enlargement of the lateral surface between two daughters at a similar stage to that in (b) in which vesicles appear to form between the IMC but do not fuse (arrows) to form the plasmalemma. IMC, inner membrane complex. Bar is 100 nm. (e) Cross section through a late stage in the second round of endodyogeny showing the four daughters located around the periphery of the organism each separated by an IMC (arrowheads) leave a central cytoplasmic mass. Bar is 1 µm. (f) Detail from an area similar to the enclosed showing the IMC to consist of two unit membranes with underlying microtubles (arrowheads). Note the interaction of the IMC with the plasmalemma (P) at the external surface to form the pellicle, but the absence of any vesicular formation associated with internal regions. Bar is 100 nm. (g) A section through the periphery of a similar stage to that in (e) showing a longitudinal section of a tachyzoite with normal organelles and thickening of the IMC at the posterior pore (arrow), but still connected along its lateral surface. Bar is 1 µm. (B) (a) Section through a control sample showing the multiple daughters formed by repeated cycles of endodyogeny arranged in a rosette. Note certain daughters still connected by the posterior end (arrow). C, conoid. Bar is 1 µm. (b) Section through a sample treated with Shld-1 for 36 hours showing a large cytoplasmic mass with numerous nuclei and the partial formation of the anterior end of certain tachyzoites. Bar is 1 µm. C, conoid; DG, dense granule; Mn, microneme; N, nucleus; R, rhoptry.

To verify the data obtained from co-localisation studies ultra-structural analysis of the phenotype observed with Rab11A_(N126I)_ was performed. Parasites stable transfected with ddFKBPRab11A_wt_ (data not shown) and ddFKBPRab11A_DN_ were inoculated on HFF cells in presence and absence of Shld-1 for 12, 24 and 36 hours. Samples were fixed and the ultra-structural appearances of the intracellular parasites analysed. In the absence of Shld-1 there was normal division of the parasites by endodyogeny resulting in two daughters connected to the residual body at the posterior end (Figure 7Aa). There were repeated rounds of endodyogeny with the fully formed tachyzoites remaining attached to the residual body by their posterior ends resulting in a rosette-like appearance by 24 and 36 hours (Figure 7Ab). In contrast, the samples treated with Shld-1 exhibited an abnormal morphology and the absence of the formation of intact tachyzoites (Figure 7Ab–7Ae). At 12 hours it was possible to observe tachyzoites undergoing endodyogeny with the formation of the two inner membrane complex associated with each nucleus. The posterior growth of the IMC and the formation of the rhoptries, micronemes and dense granules and enclosure of the divided nucleus, apicoplast and mitochondrion by the posterior growth of the IMC were similar to that seen in the controls.

There were subtle differences in the later stages of IMC growth. Once it had progressed beyond the nucleus, it appeared less rigid with an irregular outline toward the posterior (Figure 7Ab). However, the major differences were associated with the final separation of the daughters. This involves loss of the mother cell IMC and the mother cell plasmalemma associating with the IMC of the daughters to form the intact pellicle of the daughters. In the outer regions, this process appeared to occur normally, but in the internal region between daughters this process appeared disrupted (Figure 7Ab). Normally, the plasmalemma invaginates around the daughters and is assisted by fusion of vesicles formed between the IMCs of the daughters resulting in two fully formed tachyzoites attached to the residual body by their posterior end (Figure 7Aa). In the Shld-1 treated parasites, vesicular formation was disrupted with an apparent inability to fuse or form in regions, where the daughter IMCs are separated due to the irregular folding of the IMC (Figure 7Ab and 7Ad). This resulted in incomplete separation with the tachyzoites still fused along their lateral surface (Figure 7Ac). However, this incomplete cytokinesis did not prevent the daughters initiating another round of endodyogeny with the apparently normal development of two new IMCs within each of the partially separated daughters (Figure 7Ac). These repeated divisions with incomplete formation of daughters resulted in complex multi-nucleated organisms with increasing numbers of incompletely formed daughters. These 2^nd^ generation daughters are often distributed round the periphery of the organism and were separated from each other by the IMC leaving a central cytoplasmic mass (Figure 7Ae). This means the IMC of the first generation daughters had been loss, probably by the same mechanism employed by the original mother cell. The second-generation daughters containing the characteristic organelles of the tachyzoite (Figure 7Af), but only at the exterior surface did the plasmalemma interact with the IMC to form the typical pellicular structure (Figure 7Ae–7Ag). The IMC appeared normal consisting of two unit membranes with underlying microtubules (Figure 7Af) and exhibited the thickening associated with the posterior pore (Figure 7Ag). However, no vesicular formation was observed associated with the IMC on the surfaces located within the cytoplasm (Figure 7Af). At 36 hours additional divisions had occurred giving rise to complex structures often with the incompletely form daughters located round the periphery with a large central residual cytoplasmic mass. At this stage a few organisms showed partial formation of the daughter pellicle extending over the anterior third of certain daughters (Figure 7B).

## Discussion

All apicomplexan parasites undergo asexual multiplication with a start- and end-point that is always the same: an increased number of motile ‘zoites’ competent to invade new host cells. However, four variations in the process of apicomplexan asexual multiplication have been described, which differ in the number and timing of DNA replication and nuclear division and the location of daughter formation [5],[42]. Importantly, the basic process of daughter formation involving the development and growth of the IMC that is associated with the final nuclear division and apical organelle formation appears to be conserved in all variations. The major difference is that *Toxoplasma-*endodyogeny is characterised by daughter formation occurring within the mother cell cytoplasm, rather than at the surface, as seen in classical schizogony that occurs in *Plasmodium spp.* This means that only at the final stage of daughter maturation of *Toxoplasma* parasites does the mother cell plasmalemma invaginate around the daughters to form the pellicle of the mature daughter. In contrast, in *Plasmodium spp.* schizogony the nuclei move to the periphery of the mother cell and daughter formation is initiated by the appearance of the IMC adjacent to the mother cell plasmalemma and daughter cell growth is associated with budding from the surface of the mother cell, which results in the formation of the intact pellicle as the daughters grow.

Previously, in *T. gondii* we demonstrated that Rab11A has a dual role in both parasite growth and invasion [22]. In this current study we have employed three apicomplexan model organisms to analyse in detail the role of Rab11A during parasite replication. Although Rab11A can be found associated with parasite rhoptries, as described previously [21], we now show that this interaction is highly dynamic. Upon accumulation, wild type Rab11A can be readily observed in an endosomal-like compartment and at the IMC in *T. gondii*. In *P. falciparum* we observe transient co-localisation between endogenous Rab11A and MSP1 again suggestive of dynamic distribution during replication (see below). Interestingly, as soon as rhoptries are formed during the late schizont stage Rab11A significantly accumulates at rhoptries.

We provide strong evidence here that deletion of the *rab11a* gene in *P. berghei* is lethal. Consistently, induced expression of dominant negative Rab11A in *Toxoplasma* is deleterious for the parasite. In particular, we show that this small G-protein is essential for IMC maturation and for the completion of parasite cytokinesis.

During normal *Toxoplasma-*endodyogeny the plasmalemma invaginates around daughters and vesicle fusion occurs between the forming IMCs resulting in two fully formed tachyzoites attached to the residual body by their posterior end (Figure 7Aa). Upon loss of Rab11A function vesicle fusion appears disrupted and the IMCs of newly formed daughters are separated due to their irregular folding (Figure 7Ab and 7Ad), resulting in incomplete daughter separation that gives tachyzoites still fused along their lateral surface (Figure 7Ac).

Interestingly, the IMC of daughter parasites expressing a dominant negative Rab11A assembles normally through most steps of replication, with early components like GAP50 being properly inserted. However, at the final stage of replication (after biogenesis of secretory organelles), late components such as MLC, MyoA and GAP45 fail to be integrated.

**Figure 8:**
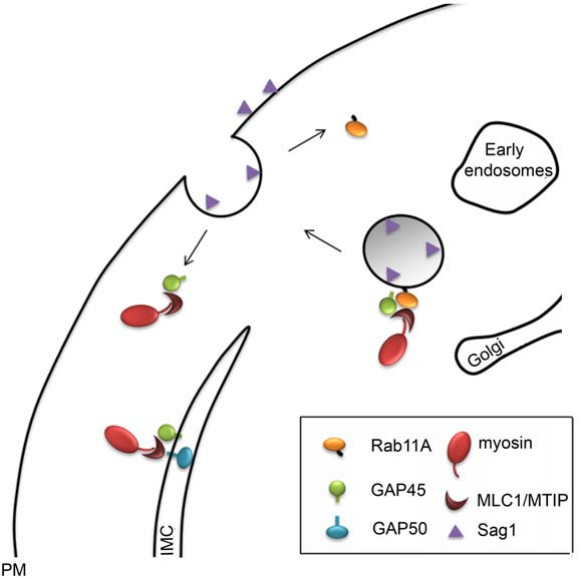
Model of Rab11A function during the late stage of parasite replication. Rab11A interacts with components of the glideosome (MLC/MTIP, GAP45 and MyoA) and transports vesicles derived from the secretory pathway to the novel synthesized plasma membrane of the daughter cells. Upon fusion of the vesicle with the PM, the complex is disassembled. Whereas Rab11A is recycled for the next round of transport, components of the proto-glideosome are freed to assemble and interact with early components of the glideosome in the IMC (like GAP50).

We argue that, similar to other eukaryotes [40], Rab11A-mediated delivery of vesicular cargo to the plasma membrane is important for completion of cytokinesis and we present a model of how Rab11A-mediated transport might contribute to IMC maturation (Figure 8). In this model Rab11A-mediated vesicular transport delivers not only new plasma membrane in between the maturing IMC of the daughter cells, but different to other eukaryotes it also delivers components of the proto-glideosome (MyoA, MLC, GAP45) [14] to the IMC. We speculate that this process not only results in maturation of the IMC, but also in its stable interaction with the plasma membrane. In support of this model we found that the immature IMC in forming daughter cells appears less rigid and is irregularly folded (Figure 7Ad and 7Af). Furthermore, the major surface antigen SAG1, which is normally anchored in the plasma membrane via its GPI moiety, can only be found in patches at the plasma membrane of the mother cell. A significant amount of SAG1 can be detected inside the parasite with a vesicular-like pattern that is consistent with partial accumulation in endosome-like compartments. We thus speculate that apicomplexan Rab11A transports vesicles derived from endosome-like compartments, similar to its known function in other eukaryotes [43],[44]. At this point we do not know if plasma membrane from the mother cell is recycled, or synthesised de novo and transported via the Rab11A-mediated secretory pathway to the furrow between mature daughter cells to complete cytokinesis.

Similarly, in *P. falciparum* we observed transient co-localisation between Rab11A and MSP1 suggestive of dynamic traffic of newly forming plasma membrane, as the IMC is forming. We have shown that *P. falciparum* Rab11A directly interacts with the myosin light chain (MLC1/MTIP), which therefore links Rab11A-mediated vesicular transport to unconventional myosins. Although several myosins exist in apicomplexan parasites (10 in *T. gondii* and 6 in *Plasmodium*) [35] only few myosin light chains can be identified in the respective genomes with only one in *Plasmodium spp.* Although MLC1/MTIP has been initially described as the light chain for MyoA [36],[37], it is plausible that several other myosins require MLC1/MTIP in order to function as a motor proteins. However, our observation that expression of DN-Rab11A alters the sub-cellular distribution of MyoA and GAP45, but not GAP50 supports a role of the motor complex known as the glideosome [14],[45],[46] in replication of the parasite. Interestingly, a recent study in *P. falciparum* demonstrated that MTIP is a substrate of CDPK1 and that kinase inhibition results in a developmental arrest at the schizont stage [47]. Based on the results presented here, we would suggest that CDPK1 inhibition and failure to phosphorylate MTIP generates a cytokinesis block.

In order to demonstrate a role of MLC1/MTIP in replication of the parasite we generated *T. gondii* parasites that strongly over-express the myosin tail of MyoA in an inducer-dependent manner. We reasoned that augmentation of MyoA tail might result in strong interaction with MLC1 and therefore, generate a MLC1 KO phenotype. Analysis of this phenotype showed that these mutant parasites are severely defective in host cell invasion and completely blocked in replication, strongly supporting a role of MLC1 and possibly MyoA in replication.

However, as mentioned above, we can't rule out that additional myosins other than MyoA might be required for replication, since MLC1/MTIP could be associated with different motors. Interestingly, over-expression of the second unconventional myosin MyoB also results in a replication-defect phenotype, causing an increase in residual bodies, which indicates a function for MyoB late in replication [9].

In summary, we demonstrate here that Rab11A interacts with MLC1/MTIP and that this association is important for completion of cytokinesis, as ablation of either Rab11A, or MLC1 function results in a specific effect on IMC organisation and cytokinesis. Of note, are our observations made here with intracellular *Toxoplasma*, where ablation of Rab11A function did not appear to have any effect on micronemes, rhoptries and dense granules during replication of the parasite. This might suggest that the observed effect on invasion upon expression of Rab11A_(N126I)_ [22], or ddFKBPMyoAtail (this report) is not due to deregulated secretion of content of these apical organelles, but rather an effect on the glideosome. Future experiments will be required to dissect the molecular mechanisms regulated by Rab11A that appear necessary for successful parasite invasion of host cells.

## Materials and Methods

### *T. gondii* culture and transfections

#### Parasite cell lines and selections

*T. gondii* tachyzoites (RH *hxgprt*–) were grown in human foreskin fibroblasts (HFF) and maintained in Dulbecco's modified Eagle's medium (DMEM) supplemented with 10% fetal calf serum, 2 mM glutamine and 25 µg/ml gentamicin. To generate stable transformants, 5×10^7^ freshly released RH*hxgprt*– parasites were transfected and selected in presence of mycophenolic acid and xanthine as previously described [48]. The selection based on pyrimethamine and chloramphenicole resistance were achieved as described previously [49],[50].

### *P. berghei* in mice and transfections

*P. berghei* (NK65) was grown in female Swiss-Webster (CD1) mice obtained from Charles River Laboratories. All animal work was conducted in accordance with European regulations. Transfection was performed using the Nucleofector device (Amaxa GmbH). 2.10^7^ purified *P. berghei* (NK65) mature schizonts were mixed with 5 µg of linearized targeting plasmid (excised from vector pDHFRΔ11a by *Hin*dIII/*Eco*RI digestion) and 100 µl of Human T cell Nucleofector solution (Amaxa GmbH). Parasites were transfected using the electroporation program U-033 available in the Nucleofector device and injected intravenously into naïve SW recipient mice. Drug resistant parasites were selected by pyrimethamine treatment using standard procedures [51].

### Generation of constructs

#### Generation of *PbΔrab11a*

For targeted deletion of the *Pbrab11a* genomic locus by replacement, two DNA fragments (494-base pair (bp) 5′-fragment and 523-bp 3′-fragment) were amplified using *P. berghei* NK65 genomic DNA as a template. The set of primers were PbRab11a-5′UTR-For (GTTAAAAAAAGCTTTTAGGTAATTATAA; *Hin*dIII site is underlined) and PbRab11a-5′UTR-Rev (CCTGGACTGCAGGTTTGATTTCC; *PstI* site is underlined), PbRab11a-3′UTR-For (GCAAATTTTGGGTACCAAATTATAAC; site *KpnI* is underlined) and PbRab11a-3′UTR-Rev (CCTTTGCATATGCAGAATTCGGGA; *Eco*RI site is underlined). Cloning into the plasmid pDEF-hDHFR targeting vector resulted in plasmid pDHFRΔ11a.

#### Rescue and generation of *PbΔrab11a-GFPrab11a* clones

The pDH-GPF11a plasmid was made from a two-step procedure: first *Pbrab11a* fragment was PCR amplified with the primers PbCDS11a-For ( GAGCTCATGTCAATGAAAGAGGATTATTACGA; *SacI* site is underlined), and PbCDS11a-Rev ( AAGCTTCGGGAGTTGTTATATTACTGAAAAT; *Hin*dIII site is underlined) using *P. berghei* cDNA as template, the 5′-UTR fragment was PCR amplified with the primers Pbrab11a-5′UTR-KI-For (GTATAAGCTTTATATTTTGTATATTT; *Hin*dIII site is underlined) and Pbrab11a-5′UTR-KI-Rev (GAAATGTCGACATATGTAGAAG; *SalI* site is underlined) and the *GFP* gene was PCR amplified with the primers GFP-For (CGCGCGGTCGACATGAGTAAAGGAGAAGAAC; *SalI* site is underlined) and GFP-Rev (CCCGGGGAGCTCTTGTTTGTATAGTTCATCCA; *SacI* site is underlined and the stop codon was mutated). Each fragment was cloned using the TA Cloning Simplifies PCR Cloning kit (Invitrogen), the plasmids were digested with the appropriate enzyme and cloned into the pDEF-hDHFR targeting vector resulted in plasmid pDHFR-GFP-Rab11A.

Transfection was performed using the Nucleofector device (Amaxa GmbH). 2.10^7^ purified *P. berghei* mature schizonts were mixed with 5 µg of *rab11a* DNA fragment (excised from vector pDHFRΔ11a by *Hin*dIII/ *Eco*RI digestion) 5 µg of pDHFR-GFP-Rab11A and 100 µl of Human T cell Nucleofector solution (Amaxa GmbH). Two clones were obtained by limited dilution series and verified by PCR analysis.

#### Constructs for *T. gondii*

The constructs for generation parasites expressing ddFKBP-tagged Rab11A fusion proteins (DD-Rab11A) [22], YFP-MyoA [52], YFP-GAP50 [14] and IMC-YFP [53] have been described previously. The DD-mCherry-Rab11a fusion vectors were cloned in two steps. First the coding region of mCherry was amplified using Oligo Cherry-s (5′-GCGGCATGCTCGCGGCGGCGGCGATGGTGAGCAAGGGCG) and Cherry-as (5′-GCGGCATGCCCCTAGGCTTGTACAGCTCGTCCATGCCGCCG) and inserted into the SphI site of the vector p5RT70DDmyc-YPT1 [22]. Subsequently the coding region of YPT1 was exchanged for Rab11A (wt/N126I) via *NsiI* and *PacI*. The resulting vectors were termed p5RT70DDmCherrymycRab11a(WT/N126I) HX.

For expression of ddFKBP-MyoA_tail_ the tail region of TgMyoA was amplified using oligonucleotide MyoA-tails (5′-GCCATGCATATTCAGAGAGAATGCCTTTCTTC) and MyoA-tails (5′- GCCTTAATTAAAACGCCGGCTGAACAGTCG). The resulting fragment was inserted into the NsiI and PacI sites of vector p5RT70DDmycMyoA-HX [22] , resulting in an exchange of MyoA for MyoA_tail_.

#### Constructs for expression of recombinant proteins

To make C-terminal PfRab11A-6-His, and N-terminal GST-PfMTIP constructs for expression in *E. coli*, the CDS of *Pfrab11a* and *PfMTIP* were PCR amplified from the cDNA of *P. falciparum* (3D7) and cloned respectively into *Bam*HI/*Xho*I site of the *E. coli* expression vector pET-21a (Novagen) and pGX6p-1 (GE Healthcare, UK). Similarly, C-terminally His-tagged constructs of PfRab5C and PfRab7 were made. All DNA sequences were verified.

### Protein expression in *E. coli*

All constructs were transformed into BL21-CodonPlus (DE3)-RIL strain (Stratagene). LB media contained 34 µg/ml chloramphenicole and 100 µg/ml ampicillin. Cells were grown at 37°C to an absorbance at 600 nm of an approximately 0.6. Proteins expression were induced by adding 0.2 mM IPTG for PfRab11A and 1 mM IPTG for PfMTIP and incubating respectively the cultures overnight at 20°C and 3 hours at 37°C. Cells were harvested by centrifugation at 5500×g for 20 min.

Harvested cells were re-suspended in urea buffer (6 M) supplemented with protease inhibitor cocktail (Roche) for PfRab11A-His and in PBS 1×, 1% Triton100× and 1 mM EDTA for PfMTIP then stored at −80°C.

His-tagged proteins purified on Ni-NTA agarose (Qiagen) in the cases of PfRab11A, PfRab5C, PfRab7 and in the case of PfGST-MTIP on Glutathione Sepharose™ 4B beads (GE Healthcare, UK).

### Serum and immuno-purification

The recombinant proteins were used to raise anti-sera in rabbits using standard procedures of Eurogentec, Belgium. The serum was applied to a protein G column (Hitrap, GE Healthcare, UK), washed, and eluted with 100 mM glycine, pH 2.5. The eluate was immediately neutralized with 1 M Tris-HCl, pH 9, and passed through an exchange buffer column (HiTrap Desalting, GE Healthcare, UK).

### Immunofluorescence

For immunofluorescence analysis of *Plasmodium falciparum* (clone 3D7) thin smears of parasites were air-dried and fixed using 3% paraformaldehyde in phosphate buffered saline (PBS) for 20 min at room temperature. Cells were permeabilised with 0.1% Triton X100 in PBS for 10 min followed by blocking in 3% BSA in PBS overnight at 4°C. Slides were incubated for 1 h with different antibody combinations: rabbit anti-PfRab11A (1∶500), mouse anti-RhopH2 (1∶1000), mouse anti-MSP1 (1∶500) and mouse anti-GAP45 (1∶1000). The slides were washed four times and incubated with AlexaFluor 488 anti rabbit IgG antibodies (1∶4000, Molecular Probes) and AlexaFluor 594 anti-mouse IgG antibodies (1∶4000, Molecular Probes Inc). Samples were examined under an epifluorescence microscope (Leica, France) with a cooled charge-coupled device (CCD) camera (Micromax, France). Images were acquired with MetaMorph (Universal Imaging, USA) and processed with MetaMorph, National Institutes of Health (NIH) image (rsb.info.nih.gov/ nih-image/) and Photoshop (Adobe Systems Inc., USA).

For immunofluorescence of *T. gondii* HFF cells grown on cover slips were inoculated with parasites in absence of Shld-1 for 4 h to allow efficient invasion. Stabilization of the respective ddFKBP-tagged protein was induced by adding 1 µM Shield- 1 for 16 hours. Cells were fixed either with −20°C cold methanol (10 minutes) or 4% paraformaldehyde (20 minutes). Fixed cells were permeabilized with 0.2% Triton X-100 in PBS for 20 minutes and blocked in 2% bovine serum albumin in PBS for 20 minutes. Staining was performed using different sets of primary antibodies for 60 min and followed by Alexa-Fluor-594-conjugated goat anti-rabbit or Alexa-Fluor-488-conjugated goat anti-mouse antibodies for another 60 min, respectively (Molecular Probes). Z-stack images of 0.15 µm increment were collected on a PerkinElmer Ultra-View spinning disc confocal Nikon Ti inverted microscope, using a 100× NA 1.6 oil immersion lens kindly provided by the Nikon Imagine Centre, Heidelberg, Germany. Deconvolution was performed using Huygens Deconvolution Software (http://www.svi.nl). Images were further processed using ImageJ 1.34r software.

### GST pull down and Western blotting

For GST pull-down experiments, 25 µg of recombinant proteins PfRab5C-His, PfRab7-His and PfRab11A-His were incubated with glutathione–Sepharose 4B (Amersham Pharmacia Biotech) coupled to 6 µl of GST or GST fusion (approximately 3 µl of bead volume) overnight at 4°C, followed by four washes in PBS (0.1% Triton X-100). These samples were then washed, and immunoprecipitated proteins were eluted by boiling in 25 µl Laemmli sample buffer (Sigma Aldrich) and electrophoresed into 4–15% SDS-polyacrilamide gel (Biorad) and transferred onto nitrocellulose. The blots were incubated with the rabbit polyclonal anti-histidine, diluted 1∶1000 (Santa-Cruz). The secondary antibody was anti-rabbit horseradish peroxidase-conjugated secondary antibody (Sigma Aldrich) and used in a 1∶5000 dilution. Immunoblots were developed by chemiluminescence using ECL (Pierce).

For Immunoblot assays on *T. gondii* parasites, Intra- or freshly lysed extracellular parasites were incubated in culture media in the absence or presence of 1 µM Shld-1 and incubated as indicated. Subsequently parasites were harvested and washed once in ice cold PBS. SDS – PAGE and Western Blot analysis were performed as described previously [54], using 6–12% polyacrylamide gels under reducing condition with 100 mM DTT. Per experiment an equal number of parasites were loaded. For detection monoclonal c-myc (9E10, Sigma-Aldrich, USA), polyclonal anti-TgMyoA [52] were used. As internal control polyclonal anti-Tub1 [55] was used.

### Growth assays of *T. gondii*

The plaque assay was performed as described before [56]. Monolayers of human foreskin fibroblasts (HFF), grown in 6 well plates, were infected with 50 to 100 tachyzoites per well. After one weak of incubation at normal growth conditions (37°C, 5% CO_2_), cells were fixed 10 minutes with −20°C methanol 100%, dyed with Giemsa stain for 10 minutes and washed once with PBS. Images were taken using a Zeiss microscope (Axiovert 200 M) with a 10× objective and plaque size was compared.

### Invasion and replication analysis of *T. gondii*

Assays were performed as previously described [22]. Briefly, 5×10^6^ freshly egressed parasites were incubated for 3 hours in presence or absence of Shld-1, before inoculation on host cells. Parasites were allowed to invade for 2 hours in presence and absence of Shld-1 and subsequently three washing steps to remove extracellular parasites were performed. Cells were then further incubated for 18 hours in presence and absence of Shld-1 before fixation. The number of vacuoles representing successful invasion events was determined in 15 fields of view and the number of parasites per vacuole was determined. The number of vacuoles represents a percentage of 100% (which reflects successful invasion) in the absence of Shld-1. Mean values of three independent experiments +/−S.D. have been determined.

### Transmission electron microscopy on *T. gondii*

Samples for electron microscopy were processed using routine techniques which can be summarised as follows: Pellets were fixed in 2.5% glutaraldehyde in 0.1 M phosphate buffer, post-fixed in osmium tetroxide, dehydrated in ethanol and treated with propylene oxide prior to embedding in Spurr's epoxy resin. Thin sections were stained with uranyl acetate and lead citrate prior to examining in a Jeol 1200EX electron microscope.

## Supporting Information

Figure S1 Rab11A from different apicomplexan parasites compared to yeast, plant and Man *Plasmodium falciparum* (Pf) Rab11A is shown aligned with that of *P. berghei* (Pb), *Toxoplasma gondii* (Toxo), *Cryptosporidium parvum* (Cp), *Arabidopsis thaliana* (At), *Homo sapiens* (H), *Saccharomyces cerevisiae* (Ypt31), *Theileria annulata* (Tha) and *Babesia gibso* (Bg). Among the different parasite species, Rab11A is highly conserved, especially the putative sites within the effector domain (stars and lines). The C-terminal double-cysteine motif required for geranylgeranylation is also indicated by arrowheads. Amino acids were aligned with ClustalW. For all accession numbers, see Table S1.
(6.75 MB TIF)

Figure S2 Insertion strategy used to generate the knockout parasite, PbΔrab11a, and the GFP-Rab11A transgenic parasite. A. The construct h-DHFR-GFPRab11A is integrated upstream of endogenous Pbrab11a locus by a single crossover event in the 5′-UTR. This gives rise to *P. berghei* transgenic parasites expressing GFP-PbRab11A. B. The targeting construct (h-DHFRΔ11a) used to delete endogenous Pbrab11a. The Pbrab11a genomic locus was targeted with the linearized plasmid containing 5′- and 3′- regions of the rab11a gene (black boxes) and the human dhfr selectable marker (h-DHFR). The endogenous gene was disrupted by double homologous recombination.
(2.59 MB TIF)

Figure S3 Expression of Rab11A(N126I) has no effect on biogenesis of secretory organelles. A) Immunofluorescence analysis of parasites stably transfected with p5RT70ddFKBPmycRab11(N126I) inoculated on HFF cells and grown in presence or absence of Shld1 for 16 hours. While a specific effect can be observed on the organisation of the IMC as shown by detection of GAP45, biogenesis of micronemes (as indicated by staining with alpha-MIC2) appears to be normal. Note that Rab11A(N126I) accumulates around the IMC of the daughter cells. B) Same experiment as in A) parasites were probed with the indicated antibodies. Only parasites treated with Shld1 for 16 hours are shown. No effect on biogenesis of rhoptries, dense granules or subpellicular microtubules was obvious.
(6.84 MB TIF)

Table S1 Accession numbers of sequences mentioned in the manuscript
(0.08 MB DOC)
