# Supplementary figures and images for: Rab11A-Controlled Assembly of the Inner Membrane Complex Is Required for Completion of Apicomplexan Cytokinesis
Source: PLoS Pathog. 2009 Jan 23;5(1):e1000270. doi: 10.1371/journal.ppat.1000270 (PMC2622761; doi:10.1371/journal.ppat.1000270)

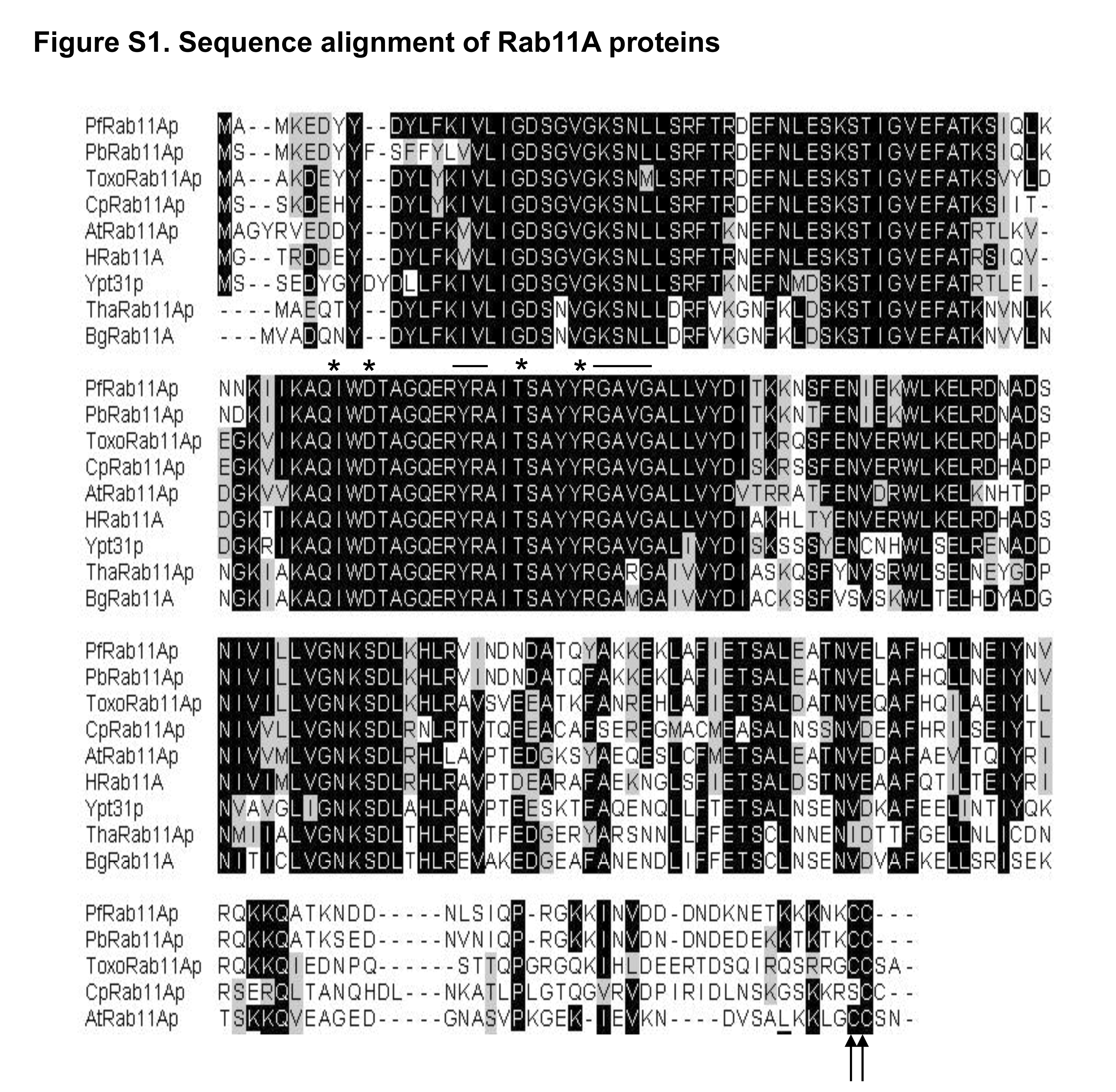

Supplement: Figure S1 — Rab11A from different apicomplexan parasites compared to yeast, plant and Man Plasmodium falciparum (Pf) Rab11A is shown aligned with that of P. berghei (Pb), Toxoplasma gondii (Toxo), Cryptosporidium parvum (Cp), Arabidopsis thaliana (At), Homo sapiens (H), Saccharomyces cerevisiae (Ypt31), Theileria annulata (Tha) and Babesia gibso (Bg). Among the different parasite species, Rab11A is highly conserved, especially the putative sites within the effector domain (stars and lines). The C-terminal double-cysteine motif required for geranylgeranylation is also indicated by arrowheads. Amino acids were aligned with ClustalW. For all accession numbers, see Table S1. (6.75 MB TIF) [file ppat.1000270.s001.tif]

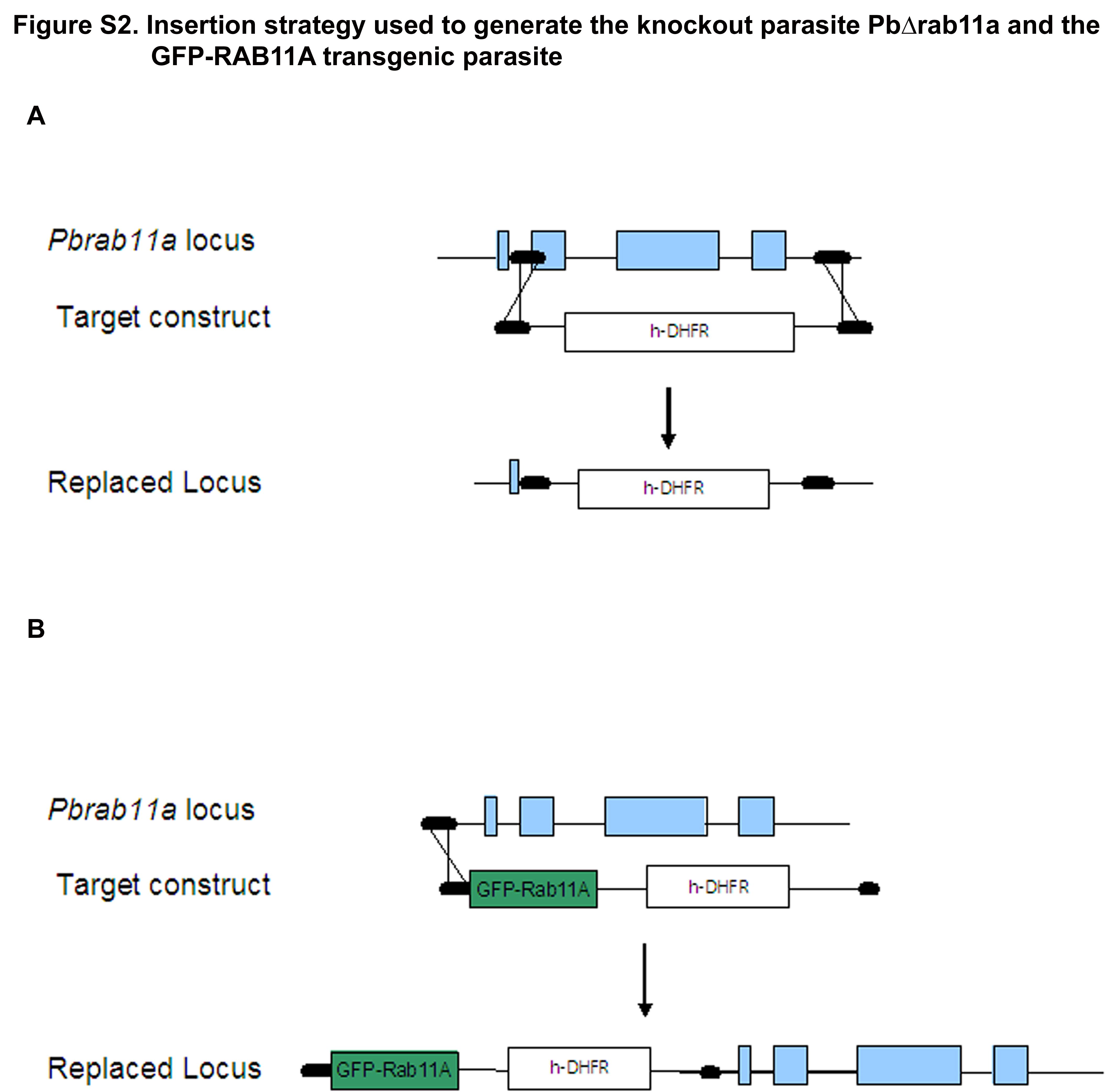

Supplement: Figure S2 — Insertion strategy used to generate the knockout parasite, PbΔrab11a, and the GFP-Rab11A transgenic parasite. A. The construct h-DHFR-GFPRab11A is integrated upstream of endogenous Pbrab11a locus by a single crossover event in the 5′-UTR. This gives rise to P. berghei transgenic parasites expressing GFP-PbRab11A. B. The targeting construct (h-DHFRΔ11a) used to delete endogenous Pbrab11a. The Pbrab11a genomic locus was targeted with the linearized plasmid containing 5′- and 3′- regions of the rab11a gene (black boxes) and the human dhfr selectable marker (h-DHFR). The endogenous gene was disrupted by double homologous recombination. (2.59 MB TIF) [file ppat.1000270.s002.tif]

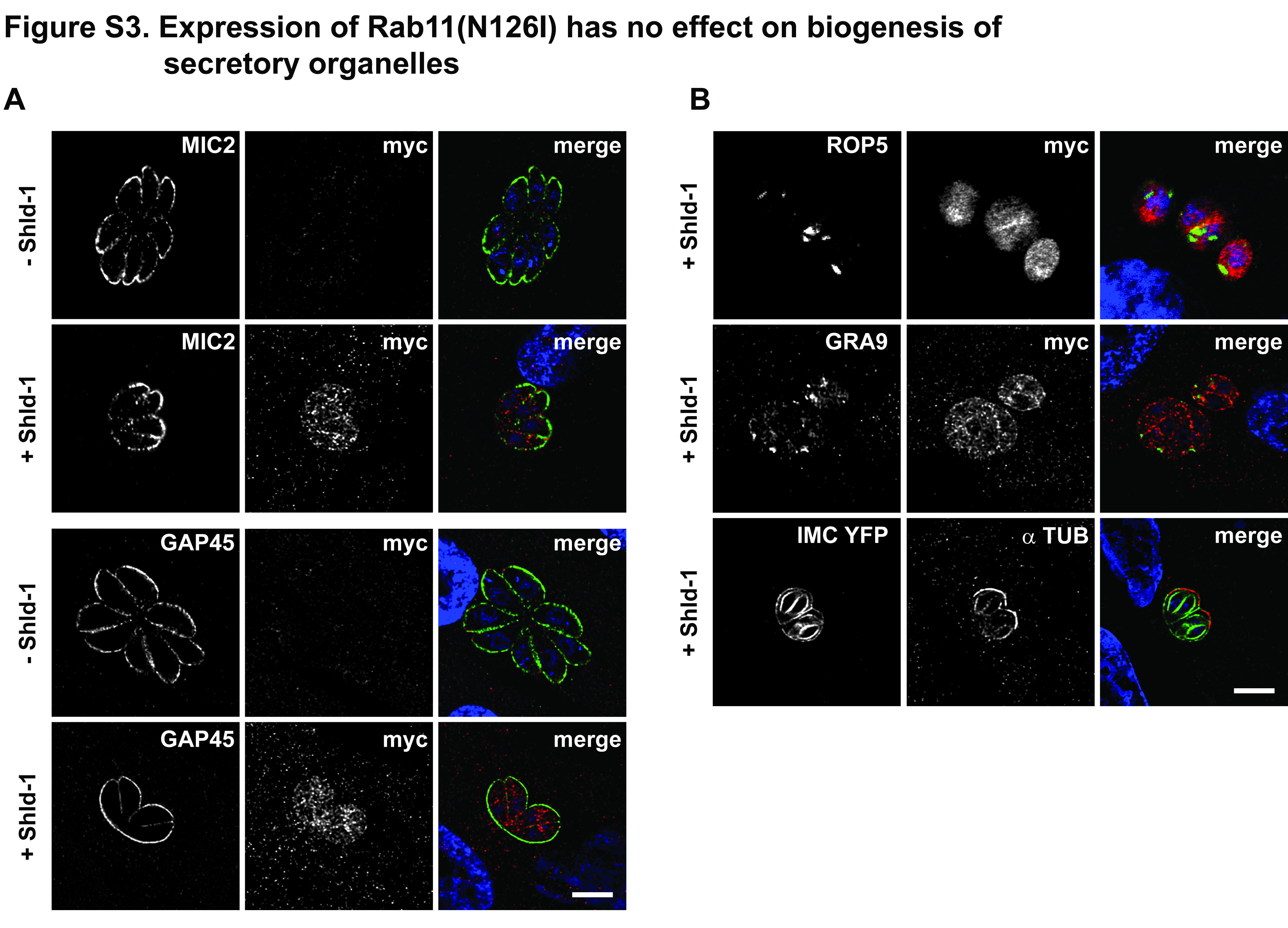

Supplement: Figure S3 — Expression of Rab11A(N126I) has no effect on biogenesis of secretory organelles. A) Immunofluorescence analysis of parasites stably transfected with p5RT70ddFKBPmycRab11(N126I) inoculated on HFF cells and grown in presence or absence of Shld1 for 16 hours. While a specific effect can be observed on the organisation of the IMC as shown by detection of GAP45, biogenesis of micronemes (as indicated by staining with alpha-MIC2) appears to be normal. Note that Rab11A(N126I) accumulates around the IMC of the daughter cells. B) Same experiment as in A) parasites were probed with the indicated antibodies. Only parasites treated with Shld1 for 16 hours are shown. No effect on biogenesis of rhoptries, dense granules or subpellicular microtubules was obvious. (6.84 MB TIF) [file ppat.1000270.s003.tif]
