# Supplementary material for: Rab11A-Controlled Assembly of the Inner Membrane Complex Is Required for Completion of Apicomplexan Cytokinesis
Source: PLoS Pathog. 2009 Jan 23;5(1):e1000270. doi: 10.1371/journal.ppat.1000270 (PMC2622761; doi:10.1371/journal.ppat.1000270)
Supplement: Table S1 — Accession numbers of sequences mentioned in the manuscript (0.08 MB DOC) [file ppat.1000270.s004.doc]

**Supplementary Table 1. Accession numbers of sequences mentioned in the manuscript**

| Name | DataBase Identifier | Acc. No. | ToxoDB Identifier | Acc. No. |
| --- | --- | --- | --- | --- |
| PfGAP50 | PFI0880c | XP_001352051 |  |  |
| PfMyoA | PF13_0233 | XP_001350147 |  |  |
| PfMTIP | PFL2225w | XP_001350849 |  |  |
| PfGAP45 | PFL1090w | XP_001350624 |  |  |
| PfRab11A | PF13_0119 | X93161 |  |  |
| TgRab11A |  |  | TGGT1_033530 | AAP57202 |
| PbRab11A | PB000861.02.00 | XM_675009 |  |  |
| PfRab11B | MAL13P1.205 | AJ879563 |  |  |
| BgRab11A | BBOV_IV006550 | XP_001610582 |  |  |
| TgRab11B |  |  | TGGT1_120910 | AAP57534 |
| TaRab11A | TA09700 | XM_948474 |  |  |
| ChRab11A | Cgd4_320 | XM_001388012 |  |  |
| PfMSP1 | PFI1475w | AAA29709 |  |  |
| TgMCL1 |  |  | [TGGT1_013010](http://beta.toxodb.org/toxo/showRecord.do?name=GeneRecordClasses.GeneRecordClass&project_id=ToxoDB&primary_key=TGGT1_013010) | [AAL08211](http://www.ncbi.nlm.nih.gov/entrez/viewer.fcgi?db=protein&id=16580144) |
| PfRab5C | PFA0335w | AJ420321 |  |  |
| PfRab7 | PFI0155c | AJ290938 |  |  |
| TgRhop5 |  |  | [TGGT1_042710](http://beta.toxodb.org/toxo5.0/showRecord.do?name=GeneRecordClasses.GeneRecordClass&project_id=ToxoDB&source_id=TGGT1_042710) | AAZ73240 |
| TgMIC2 |  |  | [TGGT1_019450](http://beta.toxodb.org/toxo/showRecord.do?name=GeneRecordClasses.GeneRecordClass&project_id=ToxoDB&primary_key=TGGT1_019450) | AAB63303 |
| TgIMC1 |  |  | [TGGT1_116030](http://beta.toxodb.org/toxo/showRecord.do?name=GeneRecordClasses.GeneRecordClass&project_id=ToxoDB&primary_key=TGGT1_116030) | AAK39634 |
| TgGAP45 |  |  | [TGGT1_078320](http://beta.toxodb.org/toxo/showRecord.do?name=GeneRecordClasses.GeneRecordClass&project_id=ToxoDB&primary_key=TGGT1_078320) | AAP41369 |
| TgMyoA |  |  | [TGGT1_070410](http://beta.toxodb.org/toxo/showRecord.do?name=GeneRecordClasses.GeneRecordClass&project_id=ToxoDB&primary_key=TGGT1_070410) | AAC47724 |
| TgGAP50 |  |  | [TGME49_019320](http://beta.toxodb.org/toxo/showRecord.do?name=GeneRecordClasses.GeneRecordClass&project_id=ToxoDB&primary_key=TGME49_019320) | AAT07037 |
| TgSAG1 |  |  | [TGME49_033460](http://beta.toxodb.org/toxo/showRecord.do?name=GeneRecordClasses.GeneRecordClass&project_id=ToxoDB&primary_key=TGME49_033460) | P13664 |
| TgM2AP |  |  | [TGME49_014940](http://beta.toxodb.org/toxo/showRecord.do?name=GeneRecordClasses.GeneRecordClass&project_id=ToxoDB&primary_key=TGME49_014940) | EEB03675 |
| Ypt31 |  | NP_011305 |  |  |
| HRab11A |  | NM_004663 |  |  |
| AtRab11A |  | NM_125479 |  |  |
